# Inflammatory bowel disease and hearing loss: A study of Mendelian randomization

**DOI:** 10.1097/MD.0000000000044863

**Published:** 2025-09-26

**Authors:** Xiaoyan Xia, Xueyu Wei, Lu Wang, Jing Deng, kunlin Pu, Hongmei Song

**Affiliations:** a College of Clinical Medicine, Chengdu University of Traditional Chinese Medicine, Chengdu, Sichuan Province, China; b Department of Otorhinolaryngology, Affiliated Hospital of Chengdu University of Traditional Chinese Medicine, Chengdu, Sichuan Province, China.

**Keywords:** autoimmunity, Crohn disease, hearing loss, inflammatory bowel disease, Mendelian randomization, ulcerative colitis

## Abstract

This study explored the intriguing causal relationship between inflammatory bowel disease (IBD) and risk of hearing loss (HL). Here, we aimed to quantify the role of autoimmunity as a potential mediator. We pooled data from genome-wide association studies (GWAS) for ulcerative colitis (UC), Crohn disease (CD), and autoimmunity from the European IEU database and genome-wide association studies (GWAS) for HL from the Finnish database. We then applied a range of rigorous statistical methods, including inverse-variance weighted (IVW), Mendelian randomization Egger regression (MR-egger), weighted median (WME), simple mode (SM), and weighted mode (WM), to perform Mendelian randomization analysis and evaluate the causal relationship between IBD and HL risk using odds ratios (OR) and 95% confidence intervals (CI). We also conducted Cochran *Q* heterogeneity test using IVW and MR-Egger regression (MR-egger), and multiple validity tests using Mendelian randomization pleiotropy residual sum and outlier (MR-PRESSO), and sensitivity analysis using the MR_leaveoneout_plot function. *F*-values were computed to assess the presence of weak instrumental variable bias. IVW results showed that in the classification of IBD, not only CD was a risk factor for HL [OR: 1.045, 95%CI: 1.007–1.086, *P* = .019], but UC was also a risk factor for HL [OR:1.370, 95%CI: 1.027–1.828, *P* = .031]. CD was a risk factor for autoimmunity [OR: 1.005, 95%CI: 1.003–1.008, *P* < .001], UC was a risk factor for autoimmunity [OR: 1.092, 95%CI: 1.051–1.135, *P* < .001], and autoimmunity was also a risk factor for HL [OR: 2.898, 95%CI: 1.048–8.009, *P* = .040]. However, the relationship between CD and HL (*P* = .127) or between UC and HL (*P* = .083) was not mediated by autoimmunity. The risk of HL is directly increased by CD and UC. The role of autoimmunity in this process is not a mediating factor.

## 1. Introduction

Hearing loss (HL) is a deterioration in hearing acuity, typically marked by elevated hearing thresholds. HL can be divided into 3 categories: conductive, sensorineural, mixed, and functional HL. The incidence of HL has increased gradually in recent years. According to the World Health Organization (WHO), approximately 1.57 billion people worldwide had HL in 2019, which could increase to more than 2.45 billion by 2050.^[1]^ The exact cause of HL is unknown; however, common risk factors include congenital, age-related, noise exposure, chronic inflammation, and mood.^[2–5]^ In previous studies, 3 possible mechanisms have been proposed for autoimmune-related ear diseases: autoantibodies bind to type II collagen or other ear components (type II immune injury), immune complex formation leads to vasculitis (type III), and T-cell-mediated auto-reactivity to the inner ear membrane components (type IV).^[6]^ This is supported by clinical studies, Aldè ^[7]^ found that 7.1% of HL cases are related to autoimmune diseases. Fousekis et al^[8]^ found that IBD is not only associated with the ear but may also be associated with diseases of the outer ear, middle ear, and inner ear. This suggests that there is a gut-inner ear axis between IBD and HL.

Inflammatory bowel disease (IBD) is a group of nonspecific inflammatory diseases of undetermined etiology that affect the intestine, including ulcerative colitis (UC) and Crohn disease (CD). The incidence of IBD is increasing worldwide, with China having the highest incidence rate in Asia (approximately 3.44/100,000 individuals.^[9]^ Kociszewska et al^[10]^ found that IBD is a multifactorial “leaky gut” disease caused by immune system dysfunction and autoimmune-induced intestinal hyperpermeability. Moreover, the presence of an involved gut-inner ear axis was indicated, linking IBD and HL. They also found any HL (mild to severe) in 29 (38%) of 76 patients with IBD, suggesting that HL might be another extraintestinal manifestation (EIM) of IBD.

Utilizing the Mendelian randomization (MR) method, the present study sought to verify the relationship between IBD and HL, in addition to confirming whether autoimmunity plays a mediating role. Furthermore, previous MR studies^[11]^ suggested that 4 types of gut flora are associated with HL, providing microbial markers for the prevention and treatment of HL. This has some reference significance for our study but does not quantify the mediating role of mediators and the relationship between intestinal diseases and HL. We aimed to assess the causal relationship between CD, UC, and HL and to identify the causal structure by assessing the mediating role of autoimmunity in the MR Framework.

The fundamental distinction between MR and randomized controlled trials is that they are among the most widely accepted experimental methods for causal inference.^[12]^ However, they are often difficult to use directly to explore causal relationships between diseases because of the stringent and difficult conditions under which the trials are designed and implemented, as well as the need to consider medical ethical issues.^[12,13]^ MR is an alternative method for simulating a randomized controlled trial. It includes genetic variants as IVs, following the principle that alleles are randomly assigned and fixed at conception, eliminating the potential for confounding, effectively reducing bias in causality estimates, and strengthening causality inference.^[14]^ MR for mediation analysis can address all of these unmet research needs because MR is less susceptible to confounding, measurement error, and reverse causation than other observational designs. These advantages can also be applied to mediation analyses, which do not require unmeasured confounding between exposure and mediator, and between mediator and outcome, which is difficult in traditional observational experiments.^[15]^

### 1.1. MR methods

This study utilized a comprehensive array of genome-wide association study (GWAS) aggregated datasets, thereby obviating the necessity for informed consent forms. This study used different statistical methods, including inverse-variance weighted (IVW), Mendelian randomization Egger regression (MR-Egger regression), weighted median (WME), weighted mode (WM), and simple mode (SM) of the MR test to make inferences about the causal effects of IBD and HL. The SM method was used to infer a causal relationship between IBD and HL, and to assess the accuracy and stability of the results. Finally, we carried out FDR correction to ensure the reliability of the results. This study used IVW as the primary approach for MR analysis, whereas MR-Egger regression and WME were used as complementary causal inference methods. Single-nucleotide polymorphisms (SNPs) were measured individually and regressed with weights using the ratio method to obtain an overall estimate, assuming that all instrumental variables (IVs) were valid and there was no multivariate validity.^[16]^ Analyses were performed using the “TwoSampleMR” package of statistical software R4.3.2, with a test level of α = 0.05.

### 1.2. GWAS summary data

**Figure 1.**
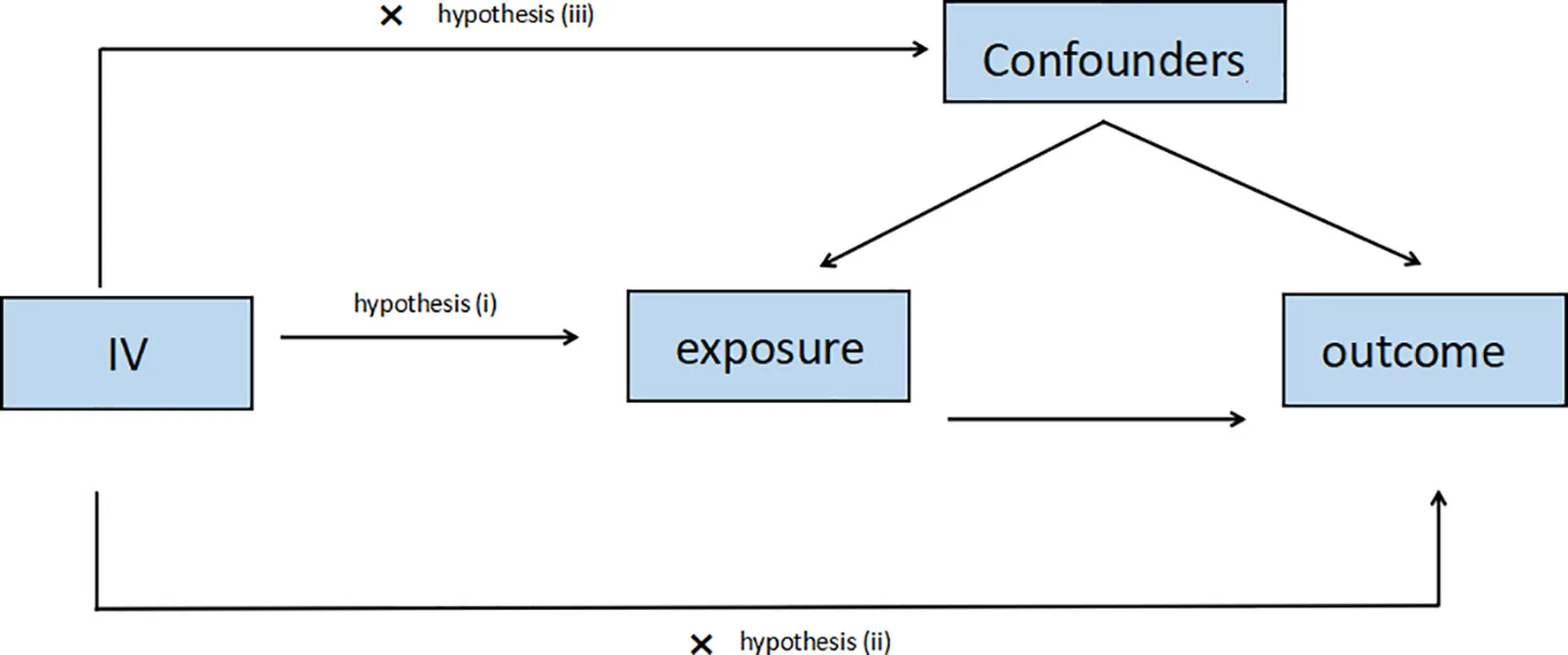
Schematic representation of the core assumptions of Mendelian randomization.

In this study, HL was used as the outcome variable, snp, which was significantly associated with CD and UC (exposure factor), was selected as the instrumental variable, and a two-sample MR analysis was used to analyze the causal associations between exposure factors and outcome variables. Table 1 lists the details of each dataset. The MR approach is used to establish a causal relationship between exposure and outcome by exploring genetic variants that exhibit strong correlations with exposure factors, such as IVs, requiring the following 3 core assumptions: genetic variants are strongly correlated with exposure (strong correlation assumption); genetic variants are not related to confounders of the exposure-outcome association (exclusivity assumption); and genetic variants can only have an impact on outcomes through exposure and not through other pathways (independent hypothesis).^[17]^ Figure 1 shows the schematic of the present analysis.

**Table 1:** Details of exposures, mediations, and outcomes.

Phenotypes	Cases	Controls	Sample size	Ancestry	Source	Pubmed ID
Crohn	5956	14,927	20,883	European	https://gwas.mrcieu.ac.uk/	26192919
Ulcerative colitis	1795	335,364	337,159	European	https://gwas.mrcieu.ac.uk/	–
Autoimmunity	–	–	469,184	European	https://gwas.mrcieu.ac.uk/	29892013
Hearing loss	4157	196,592	16,380,432	European	https://gwas.mrcieu.ac.uk/	–

### 1.3. Instrument selection

First, after standardizing the units, the calculation results are obtained. The effect size of UC is converted to the log odds ratio (log OR), while all other parameters remain unchanged. Then, quality control was performed on the IVs by screening eligible SNPs according to the following procedure: CD and UC served as exposures, and SNPs that were significantly associated with CD (*P* < 5 × 10^−8^) and UC (*P* < 5 × 10^−6^) were extracted at the genome-wide significance level. The presence of autoimmune characteristics served as a mediator, and we extracted the data using a *P* threshold of 5 × 10^−9^. To ensure that the SNPs were independent of each other, we removed linkage disequilibrium (*R*^2^ < 0.001, genetic distance kb = 10,000). The *F* statistic was calculated to assess the strength of the IVs; *F* > 10 indicates a strong IVs.^[16]^ We extracted SNPs from the resultant data and removed SNPs that were closely associated with the resultant phenotype (*P* < 5 × 10^−6^). We did not find SNPs in the resulting data, and there were no suitable proxies, so we eventually discarded them. Incompatible and palindromic alleles with moderate allele frequencies were excluded to ensure that the genetic associations reflected the same effector allele. Finally, before performing MR analysis, we performed MR pleiotropy and MR-PRESSO to identify and eliminate SNPs with potential pleiotropy. After performing the above procedures, the remaining SNPs were determined to be eligible IVs to assess the causal impact of CD and UC on HL.

### 1.4. Effect of CD and UC on HL, autoimmunity

**Figure 2.**
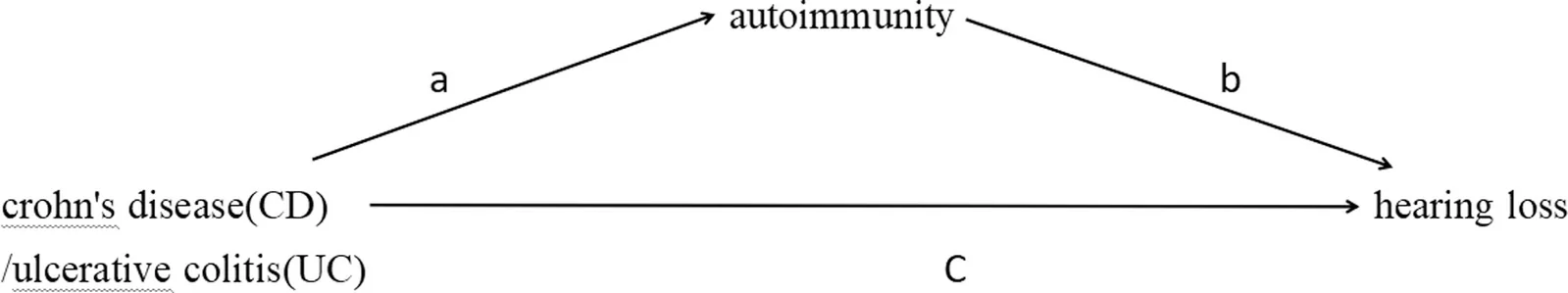
Flowchart of Mendelian randomization.

Figure 2 presents a graphical summary of the analysis. First, we used univariate two-sample MR to estimate the effect of CD or UC (exposure) on HL (outcome) (Fig. 2c), referred to as the total effect, and the effect of CD or UC on autoimmunity (mediator) (Fig. 2a). Subsequently, we evaluated the impact of autoimmunity on HL using univariate two-sample MR (Fig. 2b).

### 1.5. Mediation analysis

The goal of the mediation analysis is to explain the underlying mechanisms by which exposure affects outcomes. Conventional approaches to mediation analysis have encountered several methodological challenges, owing to the confounding effects of exposure, mediation, and measurement errors.. Mediation analysis can strengthen the causal inference of MR.^[15]^

### 1.6. Sensitivity analysis

In this study, the sensitivity of the results was assessed using MR-Egger regression,^[18]^ Cochran *Q* test, Mendelian randomization pleiotropy residual sum and outlier (MR-PRESSO test),^[19]^ and leave-one-out analysis.^[20]^ The results are presented as odds ratios (OR) and 95% confidence intervals (95% CI); both sensitivity analyses and heterogeneity tests were performed with *P* < .05 as the significance level.

## 2. Results

### 2.1. SNP-related information

Following the elimination of the incompatible allele rs12692254, and subsequent removal of confounding factors associated with autoimmunity, a total of 21 SNPs were identified as being associated with CD. Subsequent to the removal of confounding factors associated with HL, a further 48 SNPs were found to be associated with CD. Following the elimination of the incompatible alleles rs3128967, rs3128967, rs3134996, rs57791671, and rs7310615, and subsequent removal of the confounding factors related to HL, 28 SNPs remained and were found to be associated with UC. Following the elimination of confounding factors associated with autoimmunity, 24 SNPs remained, and these were found to be associated with UC. Following the removal of the incompatible alleles rs3134996, rs57791671, and rs7310615, and the subsequent elimination of confounding factors related to HL, 56 SNPs were identified as being associated with autoimmunity. All individual SNPs significantly associated with IBD and autoimmunity had *F*-statistics >10.

### 2.2. Results of two-sample MR analysis

#### 2.2.1. Effect of CD on HL and autoimmunity

The IVW results [OR: 1.045, 95% CI: 1.007–1.086, *P* = .019] suggested that CD was a risk factor for HL in the analysis of CD and HL. The MR-Egger regression method [OR: 1.004, 95% CI: 0.915–1.102], SM method [OR: 1.109, 95% CI: 0.989–1.242], WM method [OR: 1.058, 95% CI: 0.979–1.144], and WME method [OR: 1.052, 95% CI: 0.992–1.116] all supported the direction of the causality effect of IVW. In the CD and autoimmunity analyses, the IVW, MR-Egger regression, WM, and WME methods were all statistically significant, with OR (95% CI) of [OR: 1.004, 95% CI: 1.000–1.008, *P* = .018], [OR: 1.008, 95% CI: 1.002–1.014, *P* = .014], [OR: 1.004, 95% CI: 1.002–1.006, *P* = .001], and [OR: 1.003, 95% CI: 1.000–1.005, *P* = .012], respectively, and the SM method [OR: 1.001, 95% CI: 0.997–1.005] direction of causal effect was consistent with IVW.

#### 2.2.2. Effect of UC on HL and autoimmunity

The IVW results [OR:1.370, 95% CI: 1.027–1.828, *P* = .031] suggested that UC was a risk factor for HL in the analysis of UC and HL. The MR-Egger regression method [OR: 1.344, 95% CI: 0.861–2.097], SM method [OR: 1.315, 95% CI: 0.669–2.587], WM method [OR: 1.367, 95% CI: 0.902–2.072], and WME method [OR: 1.342, 95% CI: 0.921–1.955] all supported the direction of the causality effect of IVW. In the results of UC and autoimmunity analysis, the IVW method, and MR-Egger regression method were all statistically significant, with OR (95% CI) of [OR: 1.080, 95% CI: 1.034–1.129, *P* < .001], [OR: 1.128, and 95% CI: 1.046–1.216, *P* = .004], and SM method [OR: 1.030, 95% CI: 0.970–1.094], WME method[OR: 1.024, 95% CI: 0.975–1.077] and WM method[OR: 1.024, 95% CI: 0.975–1.077]direction of causal effect was consistent with IVW.

#### 2.2.3. The effect of autoimmunity on HL

The IVW method was statistically significant [OR: 1.735; 95% CI: 1.009–2.981; *P* = .045], suggesting that autoimmunity is a risk factor for HL. The MR-Egger regression method [OR: 2.506; 95% CI: 0.747–8.410], SM method [OR: 0.738, 95% CI: 0.100–5.402], WME method [OR: 0.926, 95% CI: 0.383–2.235], and WM method [OR: 0.791, 95% CI: 0.165–3.783] causal effect directions were consistent with IVW.

**Figure 3.**
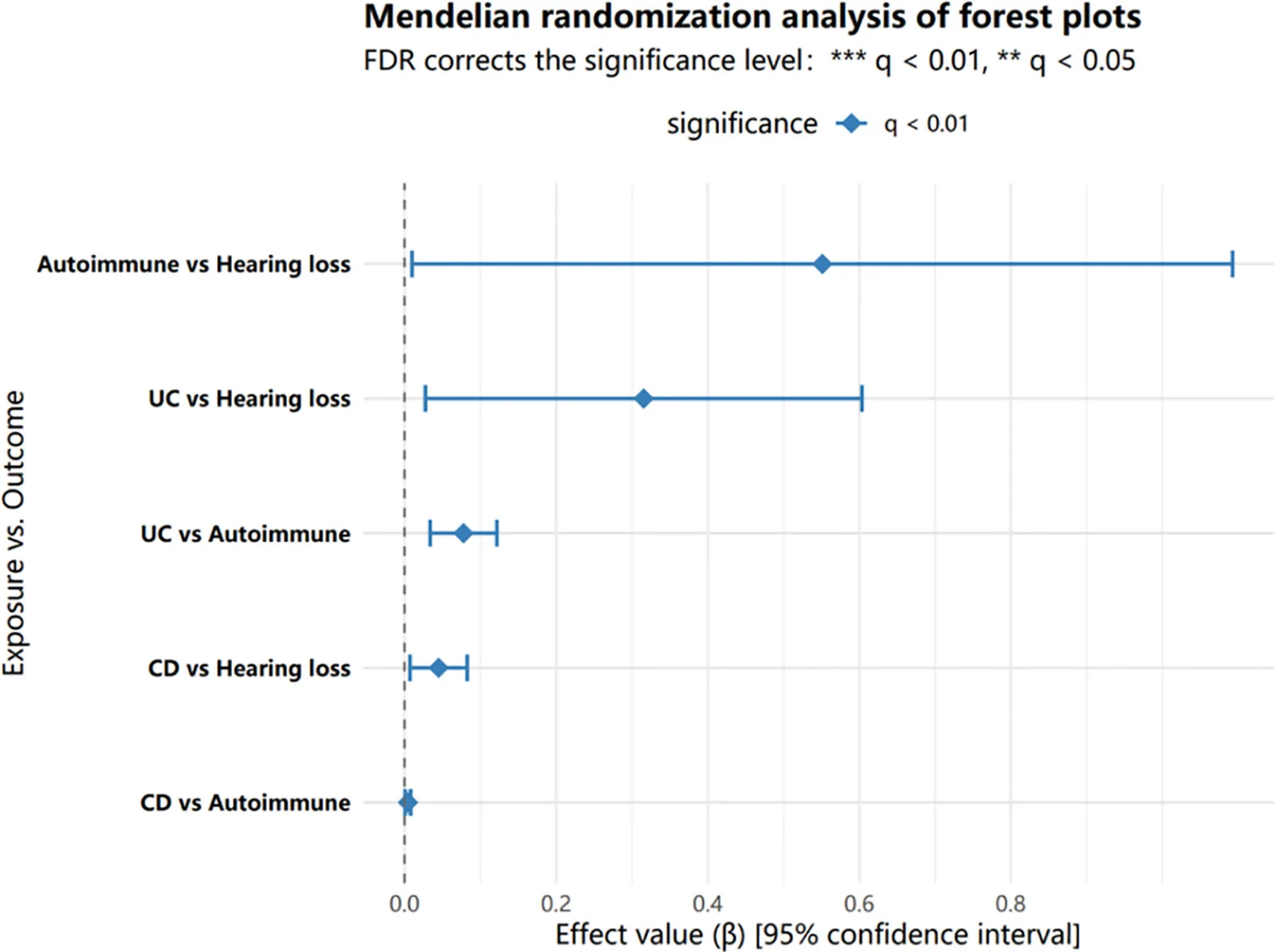
Forest plot of inverse-variance weighted results.

**Figure 4.**
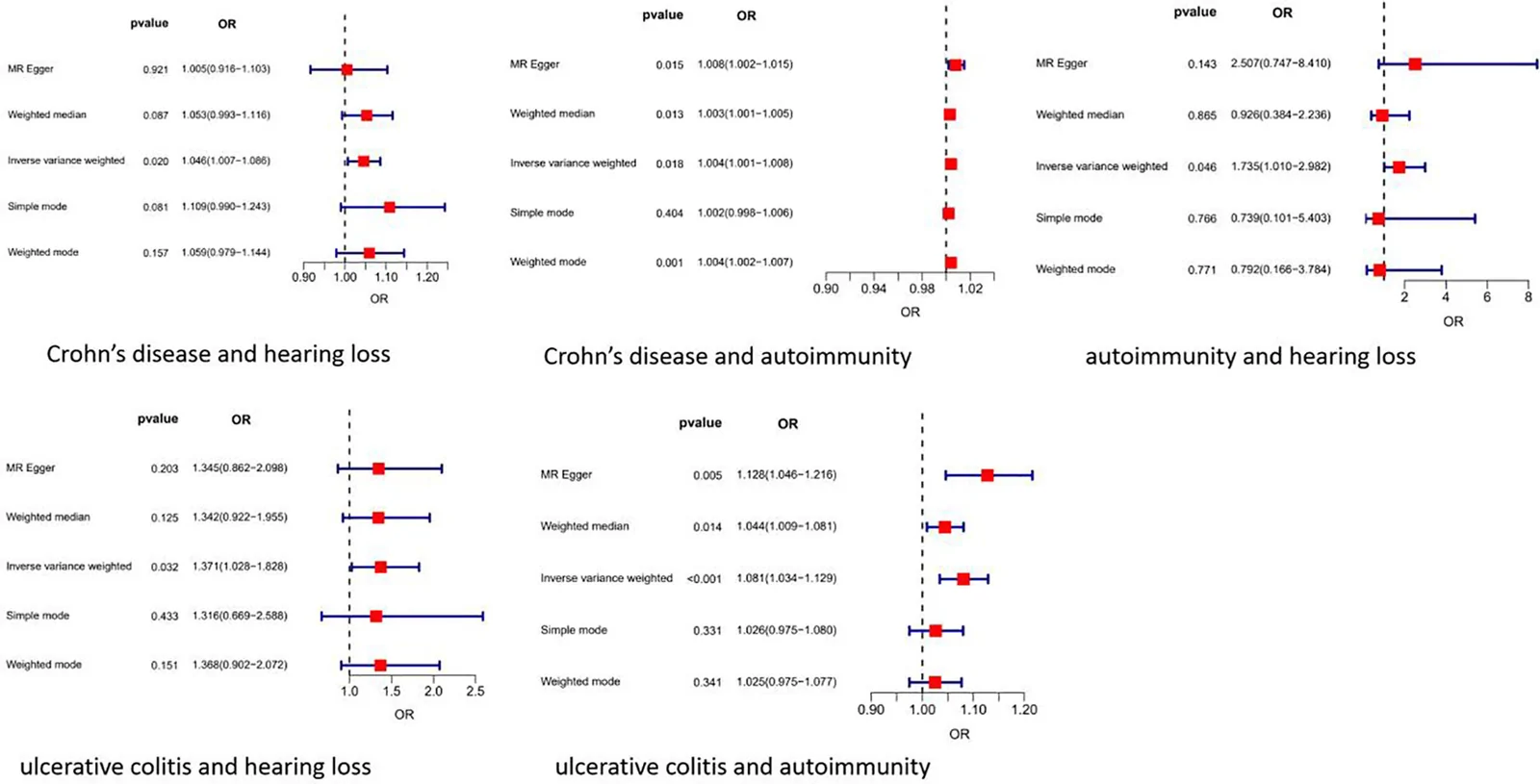
Forest plot of Mendelian randomization analysis of inflammatory bowel disease and hearing loss.

**Figure 5.**
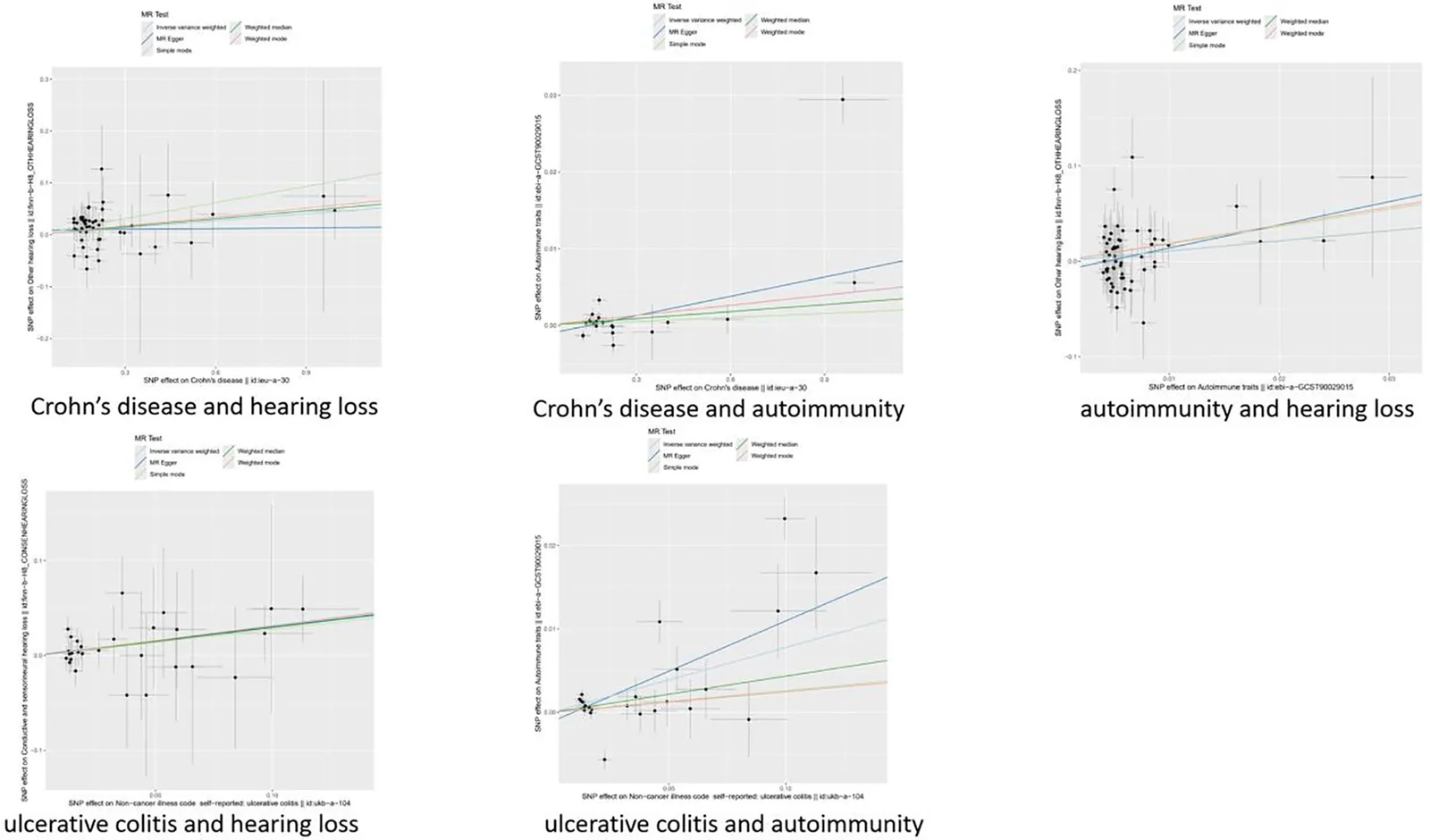
Scatter plot of Mendelian randomization analysis of inflammatory bowel disease and hearing loss.

**Figure 6.**
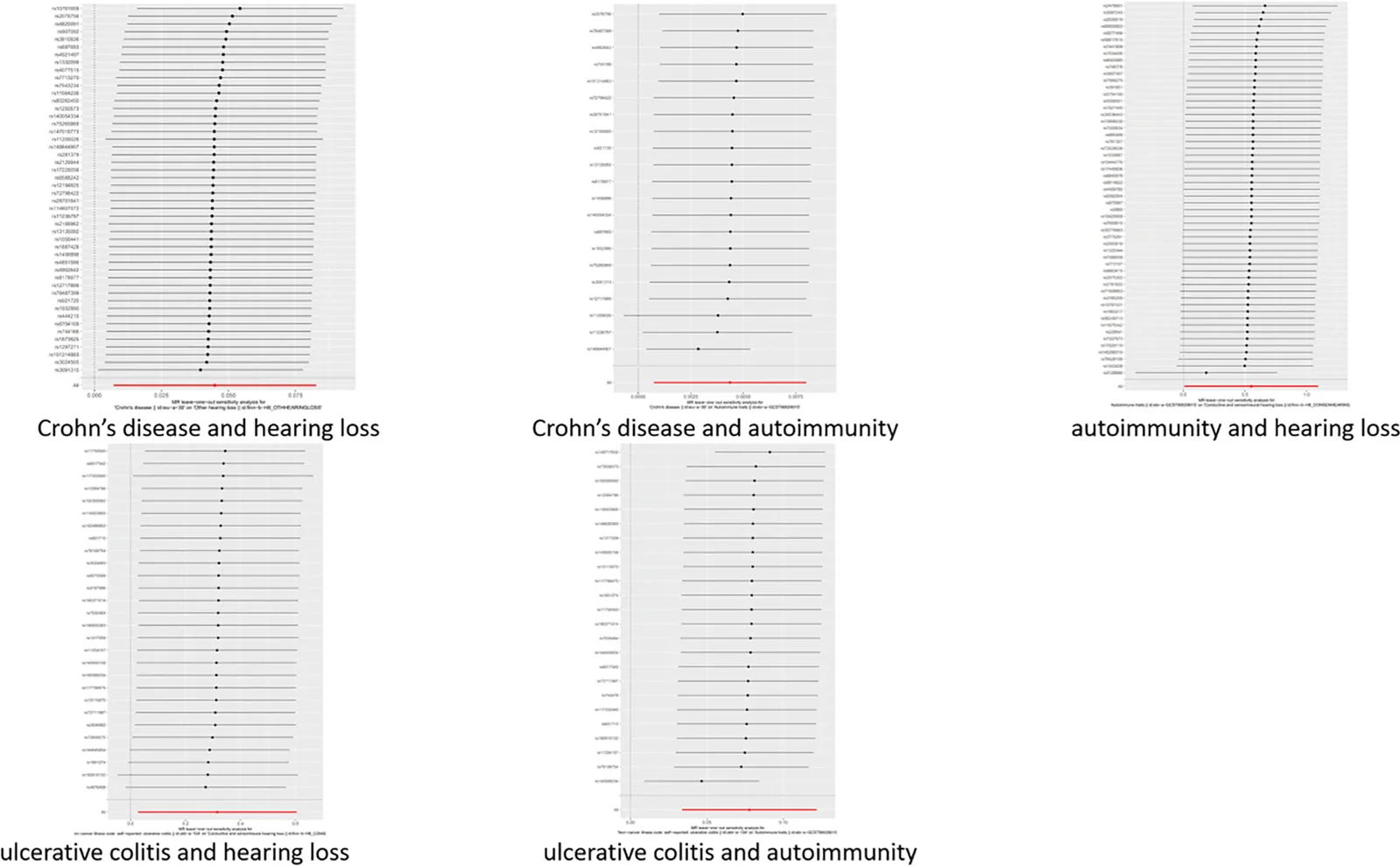
Leave-one-out method for Mendelian randomization analysis of inflammatory bowel disease and hearing loss.

**Figure 7.**
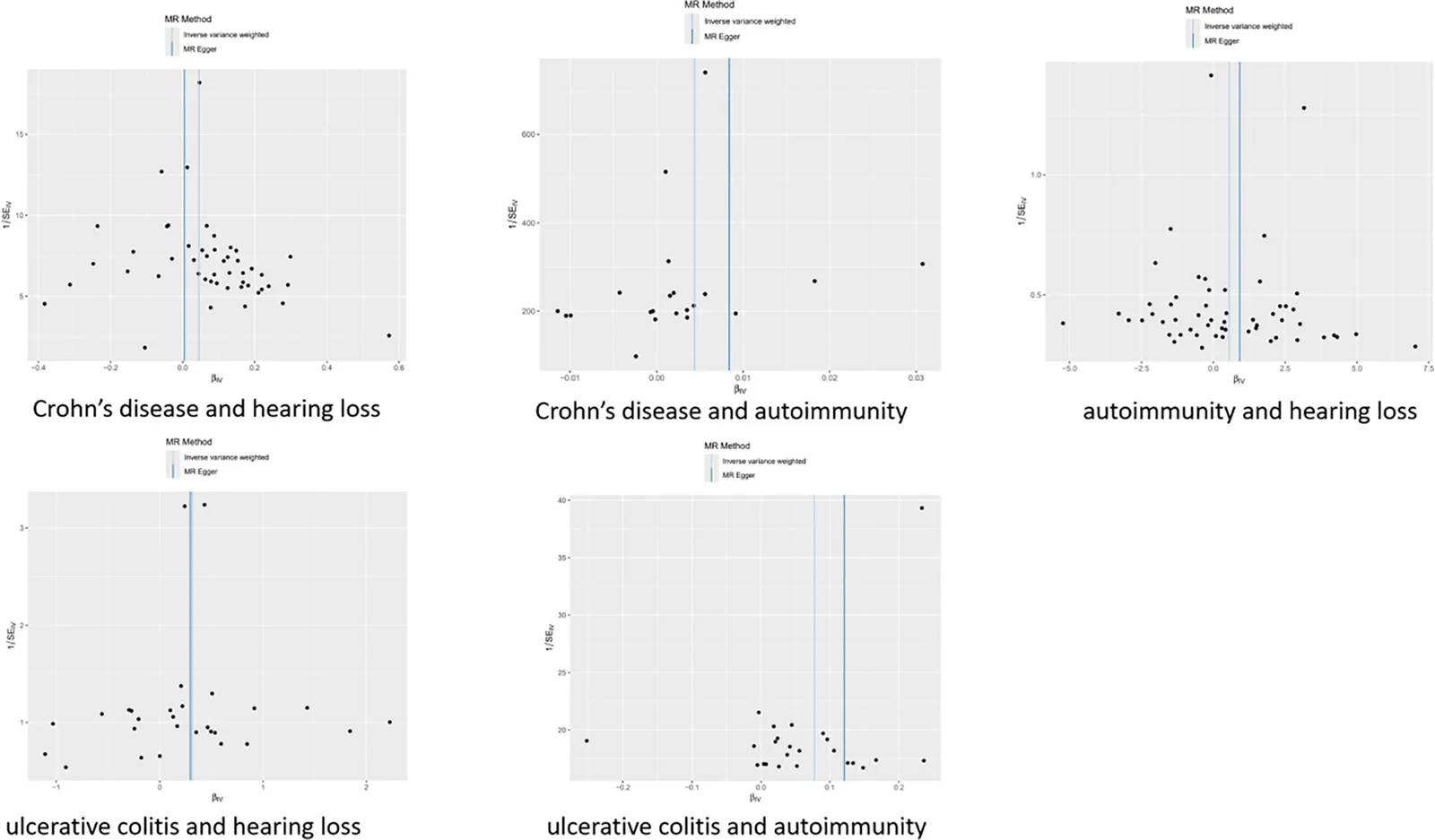
Funnel plot for Mendelian randomization analysis of inflammatory bowel disease and hearing loss.

Following the false discovery rate correction (Fig. 3), we concluded that CD was significantly associated with autoimmunity (*P* < .01) and HL (*P* < .01). Similarly, UC was significantly associated with autoimmunity (*P* < .01) and HL (*P* < .01). Furthermore, autoimmunity was significantly associated with HL (*P* < .01). Figure 4 shows the detailed results of forest plots. Figure 5 shows a scatter plot of the MR analysis. Figure 6 shows the leave-one-out method for MR analysis. Figure 7 shows a funnel plot for the MR analysis.

### 2.3. Sensitivity analysis

The IVW and MR-Egger regression methods were used to assess Cochran *Q* heterogeneity of CD and HL, with *P* = .501 and 0.495, respectively, indicating no heterogeneity. The results of the multivariate test of the Mendelian randomization Pleiotropy Test (MR_ pleiotropy_test) function yielded *P* = .395, and the MR-PRESSO method suggested ‘No outliers were identified,’ that is, there was no pleiotropy. The IVW and MR-Egger regression methods were used to assess Cochran *Q* heterogeneity of CD and autoimmunity, with *P* < .05 and *P* < .05, respectively, indicating the presence of heterogeneity. The results of the multivariate test of the MR_ pleiotropy_test function yielded *P* = .132, and the MR-PRESSO method suggested that no outliers were identified; that is, there was no pleiotropy. The IVW and MR-Egger regression methods were used to assess Cochran *Q* heterogeneity of UC and HL, with *P* = .972 and 0.980, respectively, indicating no heterogeneity. The results of the multivariate test of the MR_ pleiotropy_test function yielded *P* = .912, and the MR-PRESSO method suggested that no outliers were identified; that is, there was no pleiotropy. The results of the UC and autoimmune IVW and Cochran *Q* heterogeneity test using the MR-Egger regression method indicated significant heterogeneity (*P* < .05). The results of the polytropy test using the MR_ pleiotropy_test function yielded *P* = .189, and the MR-PRESSO method suggested” that“no outliers were identified,” that is, there was no pleiotropy. Cochran *Q* heterogeneity test for IVW and MR-Egger regression for autoimmunity and HL were *P* = .424 and *P* = .445, respectively, indicating no heterogeneity. The result of the pleiotropy test MR_ pleiotropy_test function suggested *P* = .507, whereas the MR-PRESSO regression indicated the absence of outliers and pleiotropy.

Thus, our results suggest that both between CD and autoimmunity and between UC and autoimmunity are affected by heterogeneity and not by horizontal pleiotropy. Table 2 shows the detailed results of heterogeneity and pleiotropy.

**Table 2:** Heterogeneity and pleiotropy analysis.

	Mendelian randomization Egger regression	Inverse-variance weighted method	Mendelian randomization pleiotropy test
Crohn and hearing loss	0.495	0.501	0.359
Ulcerative colitis and hearing loss	0.980	0.972	0.912
Crohn and autoimmunity	<0.05	<0.05	0.189
Ulcerative colitis and autoimmunity	<0.05	<0.05	0.373
Autoimmunity and hearing loss	0.409	0.438	0.191

### 2.4. The mediating role of autoimmunity between IBD and HL

The indirect effects of CD (β = 0.042, *P* = .083) and UC (β = 0.042, *P* = .083) on HL were not significant, indicating that autoimmunity did not play a mediating role between CD, UC and HL..

## 3. Discussion

This MR study investigated the causal relationship between IBD and HL. IBD can be divided into CD and UC, and the results of the present MR study suggest that CD and UC are risk factors for HL. The results of the GWAS analysis were consistent with clinical observational studies that found that CD and UC may be associated with HL.^[21]^ Furthermore, clinical studies have found that CD and UC are associated with localized or systemic immunopathology, and that HL may also be a manifestation of autoimmune diseases.^[22,23]^ This once again provides clinical evidence for our MR findings: UC and CD have been demonstrated to be associated with HL.

Our mediation analyses also provided genetic evidence of an association between IBD and HL. Previous studies have linked IBD and autoimmunity,^[24]^ autoimmunity and HL,^[25]^ and IBD and HL^[26]^ but have not explored whether autoimmunity plays a mediating role in IBD and HL. In this thesis, we explored the role of immunity as a mediator between IBD and HL. The findings of this study suggest that autoimmunity does not serve as a mediating factor between CD and UC, and HL. It is possible that other substances, including inflammatory markers may be responsible for the mediation.^[27,28]^

The strength of this study is that no randomized controlled trials have examined the relationship between IBD and HL. Previous observational clinical studies have been subject to several unavoidable confounders, with biased causal estimates between IBD and HL. In this study, we used SNPs as a genetic tool to minimize the bias due to confounding and reverse causality. We used GWAS data to produce highly accurate SNP effect estimates, facilitating accurate MR analyses, and thus improving the robustness of our findings.

The limitations of our study are as follows. We did not clearly classify HL, resulting in a less clear and specific relationship between IBD and HL. Despite the fact that the included data originated from European, a discrepancy remained concerning the lineage of Finnish and European. The absence of stratification in the population analysis resulted in heterogeneity in the research results. Although this study provides evidence of a causal relationship between IBD and HL, it does not explore the mechanism of action between the 2 disease entities. Autoimmunity, as a composite endpoint, has the capacity to obscure the disease-specific effects.

In conclusion, this MR study provides preliminary evidence of a causal relationship between IBD and HL risk. These findings may provide new insights into the relationship between IBD and HL, and additional studies are urgently required to validate our findings.

## Author contributions

**Conceptualization:** Hongmei Song.

**Investigation:** Xueyu Wei.

**Project administration:** Kunlin Pu.

**Resources:** Lu Wang.

**Supervision:** Jing Deng.

**Writing – original draft:** Xiaoyan Xia.

**Writing – review & editing:** Xiaoyan Xia.
